# Prognostic Modelling of Mortality in Chronic Critical Illness After Traumatic Brain Injury

**DOI:** 10.3390/jcm14228202

**Published:** 2025-11-19

**Authors:** Valery Likhvantsev, Dmitriy Kolesov, Levan Berikashvili, Elizaveta Korolenok, Mikhail Yadgarov, Kristina Kadantseva, Ivan Kuznetsov, Petr Polyakov, Artem Kuzovlev, Andrey Grechko

**Affiliations:** Research and Clinical Center of Intensive Care Medicine and Rehabilitology, Moscow 107031, Russia

**Keywords:** traumatic brain injury, chronic critical illness, intensive care units, nomograms, risk assessment, mortality

## Abstract

**Background:** Advances in intensive care have markedly improved survival from acute critical illness. Nevertheless, the subsequent trajectory of these patients is heterogeneous: while most recover and are eventually discharged, approximately 10% remain dependent on life-support systems, forming a distinct group classified as chronic critical illness (CCI). These patients experience prolonged ICU stays, high mortality, and poor long-term outcomes. Prognostication in CCI remains challenging, as traditional severity scores based on admission data seem to lose prognostic accuracy progressively over longer ICU stays. This is particularly relevant in traumatic brain injury (TBI), where patients constitute a significant proportion of the CCI population and require specialized prognostic approaches. **Objective:** To develop and validate prognostic models for in-hospital mortality in patients with TBI who progress to chronic critical illness, comparing the performance of a traditional admission-based (left-aligned) model with a novel dynamic (right-aligned) model utilizing data from the week preceding the outcome. **Methods:** A real-world data analysis was conducted using the Russian Intensive Care Dataset (RICD v2.0). The cohort included 430 ICU admissions of adult TBI patients with a stay of ≥7 days. Multivariable logistic regression was used to develop two nomograms: one using parameters from ICU admission and another using data from 7 days prior to discharge or death. Model performance was assessed via ROC analysis, sensitivity, specificity, and predictive values. **Results:** The left-aligned model, based on admission data (coronary artery disease, multiorgan failure, CRP), showed moderate discriminative capacity (AUROC 0.720). In contrast, the right-aligned model, incorporating dynamic parameters from the pre-outcome period (lymphocyte count, platelet count, urea, CRP), demonstrated excellent predictive performance (AUROC 0.889), with 90.0% sensitivity and 98.6% negative predictive value. A high score on the right-aligned nomogram was associated with a 19.7-fold increased risk of mortality within the subsequent week. **Conclusions:** For patients with CCI following TBI, a dynamic prognostic model based on data from the immediate pre-outcome period significantly outperforms traditional admission-based models. The high negative predictive value of the right-aligned model provides a reliable tool for identifying patients with a low short-term risk of mortality, supporting a paradigm shift towards dynamic risk stratification in chronic critically ill patients.

## 1. Introduction

Advances in critical care medicine, driven by technological innovation, pharmacological progress, and the adoption of evidence-based practices, have significantly improved survival rates following acute life-threatening illnesses [1,2]. However, this success has yielded an unintended consequence: the emergence of a growing population of patients who survive the initial insult but remain dependent on life-sustaining therapies for an extended period [3,4]. This clinical syndrome, termed chronic critical illness (CCI), is now diagnosed in approximately one in every fourteen patients admitted to the intensive care unit (ICU) [5], posing a substantial and growing challenge to contemporary healthcare systems [6].

CCI is characterized by profound and persistent organ dysfunction requiring prolonged ICU support, even after the resolution of the original acute condition [7]. The syndrome carries a dire prognosis, with in-hospital mortality rates of approximately 27% and one-year mortality escalating to nearly 45% [8]. Those who survive frequently experience a catastrophic loss of functional independence, requiring long-term care in specialized facilities and suffering from a severely diminished quality of life [8,9,10,11,12].

In response to the clinical heterogeneity within this patient group, evolving models seek to refine its classification. A significant recent development is the proposal of a three-stage model of CCI progression through a “prolonged critical state”, which aims to provide a more nuanced framework for prognosis and treatment stratification compared to older dichotomous models [13,14]. Despite these conceptual advances, the pathophysiology of CCI is not yet fully elucidated but is increasingly associated with a triad of persistent hypercatabolism, inflammatory dysregulation, and immune suppression (e.g., the PICS [15] or ICS [13]), which is thought to drive the development of late-onset multiple organ dysfunction syndrome [15].

A critical and debated aspect of CCI is the diminishing influence of the initial etiology on long-term outcomes over time. Large observational studies suggest that the primary diagnosis ceases to be the primary determinant of prognosis after a median of 10 days from the acute event, though this varies from 7 to 14 days depending on the cause [16]. Consequently, this necessitates a dual approach: an initial management strategy stratified by the underlying etiology, followed by a transition to syndrome-based care as the condition evolves. Furthermore, the distinct phenotypic expression of the chronic critical illness process underscores the imperative for phenotype-specific prognostic models to guide clinical decision-making and resource allocation. This paradigm highlights the imperative to study specific patient cohorts, such as individuals with severe traumatic brain injury (TBI), who constitute 6.2% of the CCI population [5].

Therefore, the objective of this study was to develop and evaluate the prognostic accuracy of various models for predicting mortality in CCI patients with traumatic brain injury.

## 2. Materials and Methods

### 2.1. Data Acquisition

A real-world data analysis was performed using the Russian Intensive Care Dataset (RICD) v2.0. This open database, developed by the Federal Research and Clinical Center of Intensive Care Medicine and Rehabilitology, contains anonymous data from 3607 ICU (intensive care unit) admissions of 3404 patients during the 2017–2024 time period [17,18].

The cohort comprised all adult patients (aged 18 years or older) with traumatic brain injury (TBI) who were admitted to the intensive care unit and stayed there for at least 7 days. The exclusion criterion was the presence of stroke on admission. All patients received standard-of-care management in the ICU, which included optimized medical and surgical treatment.

Data were extracted using DBeaver with SQLite version 3.46.1. Information was collected for all eligible ICU admissions and included (1) demographic characteristics: age, sex, body mass index (BMI); (2) comorbidities; (3) laboratory parameters; and (4) diagnostic scale scores: Full Outline of UnResponsiveness score (FOUR); Glasgow Coma Scale (GCS). Time-dependent parameters were assessed at two points: (a) ICU admission (first value within 48 h after admission, defined as the left-aligned point), and (b) 7 days prior to ICU discharge or death (the right-aligned point).

The primary endpoint was all-cause mortality during the intensive care unit stay.

This study was conducted in accordance with the STROBE guidelines (Supplemental S1).

### 2.2. Statistical Analysis

Continuous variables were reported as medians (Me) with interquartile ranges (IQR), while categorical variables were presented as absolute counts and percentages (%). Group comparisons were made using the Mann–Whitney U test for continuous variables and Fisher’s exact test (two-sided) for categorical ones. Univariable logistic regression was performed for both left- and right-aligned points.

In order to identify independent parameters associated with mortality, variables that showed statistically significant differences in univariable analyses, along with those considered clinically relevant, were included in the multivariable logistic regression model. This model was subsequently fitted using Wald’s backward elimination method, resulting in a left-aligned nomogram [19]. The process was repeated for two separate time points, resulting in two nomograms. The left-aligned model development considered baseline parameters measured upon admission to the ICU (first time point), while the right-aligned model used parameters measured at the second time point, which was 7 days prior to the outcome.

Model performance was evaluated using receiver operating characteristic (ROC) analysis. Sensitivity, specificity, positive predictive value (PPV), negative predictive value (NPV), and risk estimates were calculated at the optimal cut-off point determined by Youden’s index.

The significance level was set at *p* < 0.05. Analyses were performed using IBM SPSS Statistics v.27 (IBM Corp., Armonk, NY, USA) and Stata/MP 18.0 (StataCorp LLC, College Station, TX, USA).

## 3. Results

**Figure 1:**
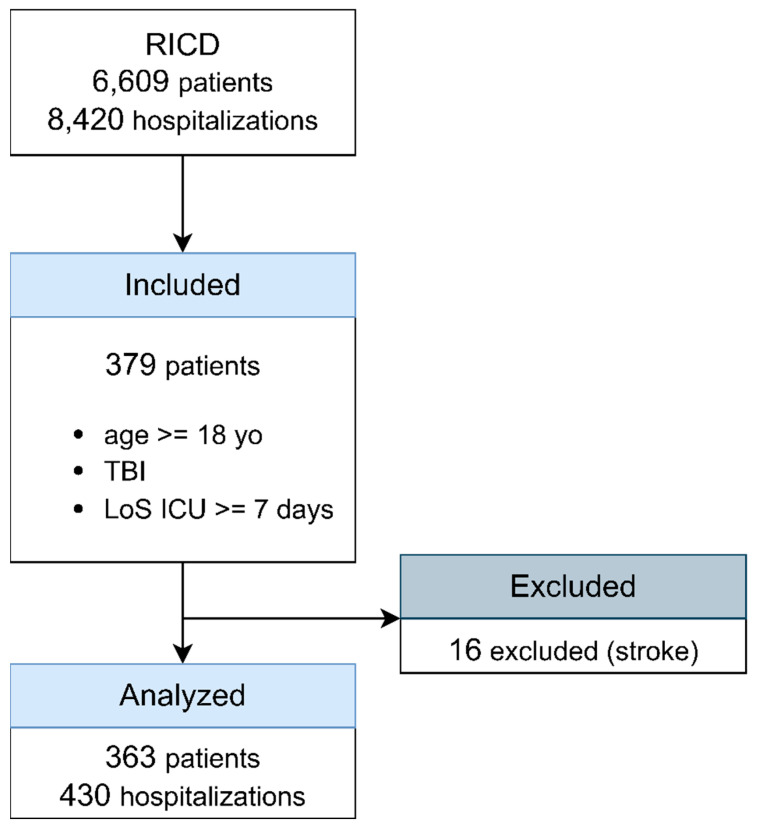
Flowchart showing selection of patients and hospitalizations. Abbreviations: LoS ICU, length of stay in the intensive care unit; RICD, Russian Intensive Care Dataset; TBI, traumatic brain injury.

The patient selection process is presented as a flowchart in Figure 1. In total, 379 patients met the inclusion criteria. After excluding patients who had suffered a stroke during their actual admission to the intensive care unit and accounting for those with repeated ICU stays, a total of 430 separate ICU admissions were analyzed.

The majority were male (315, 73.3%) (Table 1). The median age was 42.5 years (IQR 32–57), with a median BMI of 21.5 kg/m^2^ (IQR 18.8–24.7). Common comorbidities included arterial hypertension (34.2%) and coronary artery disease (11.6%). On admission, polytrauma was observed in 12.3% of patients, multiorgan failure in 65.4%, and the median SOFA score was 3 (IQR 2–5). Neurological status was assessed using the GCS and FOUR scores, with median values of 10 (IQR 8–13) and 13 (IQR 11–15), respectively. The median ICU length of stay was 39 days (IQR 24–70).

### 3.1. Left-Aligned Model

Upon admission, significant differences in clinical and laboratory parameters were observed between survivors and non-survivors (Table 1).

The results of the univariable analysis identifying admission risk factors associated with mortality are presented in Table S1. Multivariable analysis, using a left-aligned model, identified the following independent risk factors for mortality: presence of coronary artery disease (OR 2.914 [1.160; 7.316]; *p* = 0.023), multiorgan failure on admission (OR 3.878 [1.101; 13.659]; *p* = 0.035), and elevated CRP levels (OR 1.005 [1.000; 1.009]; *p* = 0.044) (Table S2).

**Figure 2:**
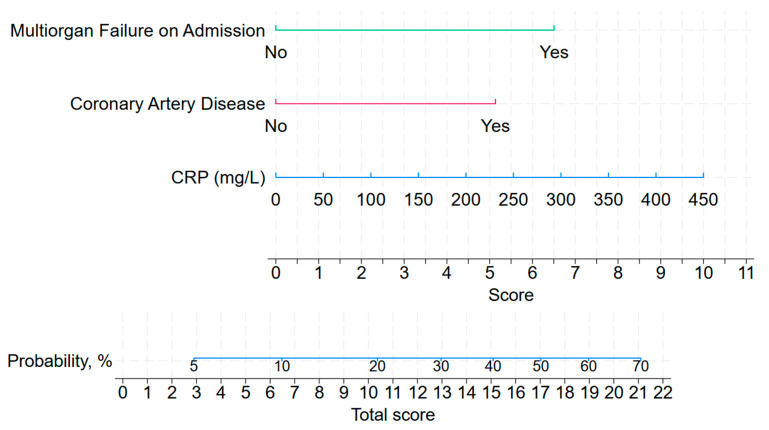
Nomogram for predicting in-hospital mortality on ICU admission (left-aligned model). Abbreviations: CRP, C-reactive protein.

Based on these predictors, a nomogram was constructed to estimate the probability of death, as presented in Figure 2.

### 3.2. Right-Aligned Model

At 7 days before ICU discharge, survivors and non-survivors also showed significant differences across multiple laboratory and clinical parameters (Table 2).

The results of the univariable analysis identifying risk factors associated with mortality within a 7-day period are presented in Table S3. In the right-aligned multivariable logistic regression model lower lymphocyte counts (OR 0.217 [0.072; 0.659]; *p* = 0.007) and lower platelet counts (OR 0.995 [0.990; 1.000]; *p* = 0.046) were associated with increased mortality risk along with higher urea concentrations (OR 1.243 [1.066; 1.449]; *p* = 0.005) and elevated CRP levels (OR 1.010 [1.001; 1.019]; *p* = 0.022) (Table S4).

**Figure 3:**
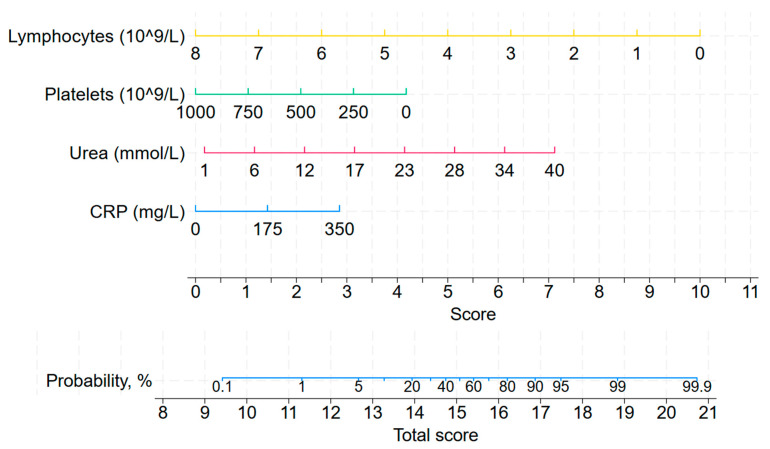
Nomogram for predicting 7-day mortality in the ICU (right-aligned model). Abbreviations: CRP, C-reactive protein.

Based on these predictors, a nomogram was constructed to estimate the probability of mortality at this time point, as shown in Figure 3.

**Figure 4:**
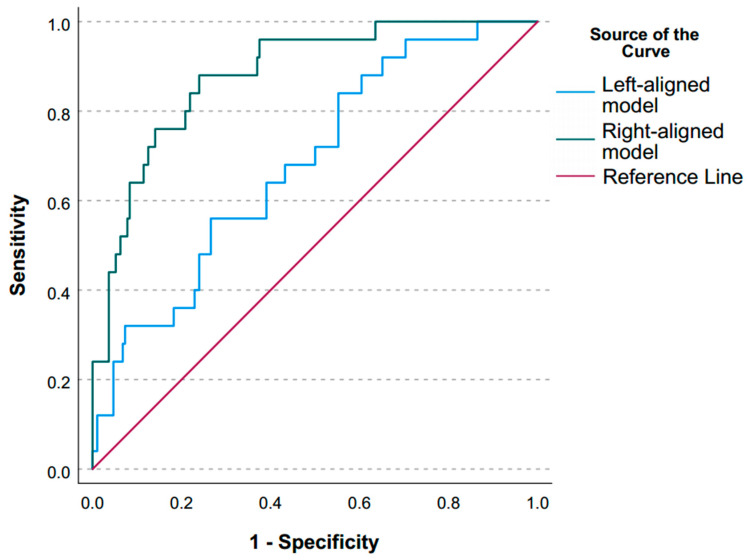
ROC-curves for left- and right-alignment models.

Both left- and right-aligned models demonstrated significant discriminatory capacity for mortality with AUROC of 0.720 (0.631; 0.808) and 0.889 (0.831; 0.947), respectively (Table S5, Figure 4). The admission score achieved a sensitivity of 58.8% and specificity of 75.7% at the optimal cut-off of 8.4. This model yielded a high negative predictive value (NPV) of 93.2%, but a modest positive predictive value (PPV) of 24.4%, indicating better ability to rule out than to confirm the prognosis of death.

By contrast, the score calculated 7 days before ICU discharge or death (right-aligned) demonstrated excellent discrimination, with sensitivity reaching 90.0% and specificity 74.9%. The optimal cut-off for the score was 12.3. The model showed a very high NPV of 98.6% though PPV remained modest (27.8%). This later score markedly improved risk stratification, showing very high odds ratio (OR 26.9, 95% CI 7.9–91.3) and relative risk (RR 19.7, 95% CI 6.1–63.3) for mortality in patients above the threshold.

The ROC-curves (Figure 4) illustrate the clear performance difference between the two models, with the right-aligned score significantly outperforming the admission-based score in predicting mortality.

## 4. Discussion

A real-world data analysis utilizing the RICD v2.0 registry identified independent risk factors for in-hospital mortality in CCI patients hospitalized with traumatic brain injury. The observed mortality rate in this cohort was 10%, with a median ICU length of stay of 39 days.

### 4.1. Key Findings

Multivariable analysis identified coronary artery disease (*p* = 0.023), multiorgan failure on admission (*p* = 0.035), and elevated CRP levels (*p* = 0.044) as independent risk factors for mortality at ICU admission. Notably, the predictors of mortality shifted when the analysis was aligned to seven days prior to death or discharge. In this model, the independent factors were lower lymphocyte (*p* = 0.007) and platelet (*p* = 0.046) counts, higher urea (*p* = 0.005) and CRP (*p* = 0.022) levels.

The model based on admission factors (left-aligned) demonstrated moderate discriminative capacity, with an AUROC of 0.720, sensitivity of 58.8%, and specificity of 75.7%. In contrast, the right-aligned model, incorporating data from the week preceding the outcome, exhibited superior predictive performance, achieving an AUROC of 0.889 with a sensitivity of 90.0% and specificity of 74.9%. Furthermore, a score of 12.3 or greater on the right-aligned nomogram was associated with a 19.7-fold increase in the risk of mortality within the subsequent seven-day period.

### 4.2. Relationship with Previous Studies

The results of this study align with previously published research. For example, Carson SS et al. demonstrated the low prognostic value of left-aligned models such as the APACHE II and SAPS II scores in predicting mortality in patients with prolonged ICU stays, with AUROC values of 0.63 and 0.69, respectively [20]. Although our left-aligned model was developed using data from chronically critically ill patients, which likely improved its predictive value to an AUROC of 0.72, its prognostic accuracy remains insufficient for clinical use.

The ProVent score, developed in 2008 to predict one-year mortality in mechanically ventilated patients, demonstrated good predictive value with an AUROC of 0.82 [21]. Although this model is technically left-aligned, its evaluation timeframe differs from typical models of this type. Rather than assessing patients at ICU admission, the ProVent score evaluates patients on day 21 of mechanical ventilation. This assessment format aligns it more closely with the right-aligned approach that accounts for dynamic changes in patient status. The inclusion of patient conditions such as platelet count, vasopressor use, and hemodialysis on day 21 of mechanical ventilation likely contributed to the model’s good performance. However, these results highlight the potential advantage of our model. Validation of this score for use on day 14 of mechanical ventilation showed reduced predictive value with an AUROC of 0.74, possibly due to this assessment timeframe moving closer to traditional left-aligned models [22].

Prognostic models such as IMPACT and CRASH are well-validated for predicting mortality in patients with acute traumatic brain injury [23]. However, their applicability to patients who have entered a state of chronic critical illness remains unconfirmed. Consequently, a direct comparison with our model, developed specifically for this CCI cohort, is not presently justified and would require prior validation of these existing scores in a similar population.

The use of artificial intelligence models analyzing dynamic changes in patient condition to predict one-year mortality also failed to demonstrate high performance with an AUROC of 0.76, despite analyzing 250 parameters [24]. The likely main reason for this result, despite assessing dynamic patient changes, was the limitation to data from only the first 7 days of mechanical ventilation, effectively making this a left-aligned approach. The use of artificial intelligence models in a right-aligned format might improve prediction accuracy, as supported by the higher AUROC value of our model compared to other regression models.

### 4.3. Significance of Study Findings and Clinical Utility

The significance of our findings lies in demonstrating the need to abandon the use of left-aligned prediction models for outcomes in chronically critically ill (CCI) patients in favor of right-aligned models. The low prognostic accuracy and sensitivity of the model based on ICU admission data may be attributed to the prolonged ICU stay (median 39 days). This finding is consistent with data showing the loss of predictive value for the initial etiology of critical illness by approximately day 10. Furthermore, these results align with the concept of chronic critical illness, wherein the connection between a patient’s long-term prognosis and their initial acute presentation becomes diminished or severed.

In contrast, the right-aligned model demonstrated excellent discriminatory power, with high sensitivity and specificity. Its superior performance is likely attributable to its alignment with the dynamic, longitudinal assessment intuitively employed in clinical practice. Crucially, this model accounts for the evolution of a patient’s clinical profile throughout the chronicity of their illness—a factor omitted by the static, left-aligned approach, hence explaining the disparity in their predictive accuracy. The association of increased CRP and urea levels with higher risk, along with the protective effect of higher lymphocyte counts, supports the role of a combined pathophysiology—encompassing persistent inflammation, hypercatabolism, and immunosuppression—in the development of late-stage organ failure and increased mortality.

Consequently, our results argue for a paradigm shift within the scientific community towards dynamic outcome prediction using right-aligned models, which is particularly vital for managing the CCI patient population. The utility of this model in real-world clinical settings lies in its ability to quantify short-term risk and delineate the clinical course of patients with chronic critical illness. These capabilities allow for therapy optimization, enhanced efficiency in clinical decision-making, and improved family communication regarding short-term mortality risk.

### 4.4. Strengths and Limitations

This study has several strengths. First, it utilizes a large, prospectively collected, real-world registry (RICD v2.0), which enhances the generalizability of our findings to clinical practice. Second, the application of a standardized data extraction protocol and adherence to STROBE guidelines strengthen the methodological rigor. Third, the right-aligned model demonstrated exceptionally high discriminatory power (AUROC 0.889) and a very high negative predictive value (98.6%), making it a clinically valuable tool for identifying patients with a very low short-term risk of mortality. In addition, the use of distinct predictor sets for the right- and left-aligned models was a deliberate feature of our study design. This approach directly reflects the different clinical questions each model addresses and, far from biasing the results, is essential for a valid comparison of their prognostic utility at different time points.

However, our findings should be interpreted in the context of certain limitations. The single-center origin of the dataset may limit the direct extrapolation of the specific prediction models to other healthcare systems and patient populations with different demographics, clinical practices, and etiologies of TBI. Moreover, these models are explicitly designed for the CCI population, and their application to acute TBI cohorts is not recommended due to undefined prognostic utility and fundamentally different predictive factors. External validation in independent, international cohorts is necessary to confirm the generalizability and clinical utility of the proposed nomograms. Additionally, while the right-aligned model performed excellently, its positive predictive value remained modest (27.8%), underscoring the challenge of definitively predicting death even in this high-risk CCI cohort and highlighting that the model is best used for risk stratification rather than absolute prognostication for individual patients. Furthermore, the lack of data on the patients’ neurological status prior to TBI is a recognized limitation, as it may confound the attribution of outcomes solely to the traumatic incident.

### 4.5. Future Studies and Prospects

This study opens several promising avenues for future research. Primarily, the developed right-aligned nomogram requires external validation in independent, preferably multinational, cohorts of patients with chronic critical illness to confirm its generalizability and calibrate its performance across diverse healthcare settings. Future work should also focus on expanding the outcome measures beyond ICU mortality to include long-term functional recovery, neurological outcomes, and health-related quality of life. Integrating the model into a dynamic, automated clinical decision support system within the electronic health record could facilitate real-time risk stratification and prospectively evaluate its impact on clinical decision-making, resource allocation, and patient outcomes. Furthermore, a deeper investigation into the biological mechanisms driving the identified late phase predictors, specifically persistent inflammation reflected by elevated CRP, immunosuppression indicated by lymphopenia, and a catabolic state marked by elevated urea, could uncover novel therapeutic targets to alter the clinical course of chronic critical illness after traumatic brain injury.

## 5. Conclusions

This study demonstrates that a right-aligned prognostic model, incorporating dynamic clinical data from the week preceding an outcome, provides superior accuracy for predicting mortality in patients with chronic critical illness following traumatic brain injury compared to traditional admission-based models: FOUR and Glasgow Coma Scale. The model’s high negative predictive value offers a reliable tool for identifying patients with a favorable short-term prognosis. These findings advocate for a paradigm shift towards dynamic risk stratification to guide clinical decision-making in chronic critically ill patients.

## Figures and Tables

**Table 1:** Baseline ICU admission parameters (survivors vs. non-survivors).

Parameter	All, n = 430	Survivors, n = 387	Non-Survivors, n = 43	*p* (Mann–Whitney/Fisher)
Sex, male	315 (73.3%)	282 (72.9%)	33 (76.7%)	0.717
Age, years	42.5 (32; 57)	42 (31; 56)	45 (34; 70)	0.088
BMI, kg/m^2^	21.5 (18.8; 24.7)	21.9 (18.8; 24.8)	20.8 (18.8; 24.7)	0.431
Comorbidity				
Atrial Fibrillation	2 (0.5%)	1 (0.3%)	1 (2.3%)	0.190
Coronary Artery Disease	50 (11.6%)	38 (9.8%)	12 (27.9%)	0.002
Valvular Heart Disease	5 (1.2%)	4 (1.0%)	1 (2.3%)	0.411
Arterial Hypertension	147 (34.2%)	129 (33.3%)	18 (41.9%)	0.309
Type 2 Diabetes	11 (2.6%)	10 (2.6%)	1 (2.3%)	>0.9
Type 1 Diabetes	4 (0.9%)	3 (0.8%)	1 (2.3%)	0.345
CKD	9 (2.1%)	5 (1.3%)	4 (9.3%)	0.008
COPD	1 (0.2%)	1 (0.3%)	0 (0.0%)	>0.9
Polytrauma	53 (12.3%)	47 (12.1%)	6 (14.0%)	0.806
Multiorgan Failure on Admission	219 (65.4%)	186 (62.6%)	33 (86.8%)	0.003
Malignant Tumor	1 (0.2%)	1 (0.3%)	0 (0.0%)	>0.9
Laboratory parameters (first 48 h in the ICU)				
RBC (10^12^/L)	3.6 (3.2; 4.1)	3.6 (3.2; 4.1)	3.3 (2.8; 3.8)	0.009
Hemoglobin (g/L)	104.0 (93.0; 118.0)	105.0 (94.0; 119.5)	94.0 (84.0; 107.0)	<0.001
WBC (10^9^/L)	9.1 (6.8; 11.5)	8.9 (6.8; 11.5)	9.5 (7.2; 11.7)	0.459
Neutrophils (10^9^/L)	6.6 (4.5; 9.0)	6.5 (4.5; 9.1)	7.0 (5.2; 8.4)	0.537
Eosinophils (10^9^/L)	0.1 (0.1; 0.3)	0.2 (0.1; 0.3)	0.1 (0.1; 0.4)	0.430
Basophils (10^9^/L)	0.1 (0.0; 0.1)	0.1 (0.0; 0.1)	0.0 (0.0; 0.1)	0.037
Lymphocytes (10^9^/L)	1.4 (1.0; 1.8)	1.4 (1.0; 1.8)	1.4 (1.0; 1.9)	0.797
Platelets (10^9^/L)	330.5 (247.0; 425.0)	332.0 (249.0; 429.0)	311.0 (220.0; 409.0)	0.259
Creatinine (µmol/L)	66.3 (53.3; 80.8)	66.3 (53.3; 80.4)	67.1 (56.0; 84.7)	0.444
Urea (mmol/L)	4.6 (3.1; 7.4)	4.5 (3.0; 7.3)	5.4 (4.0; 8.3)	0.028
Potassium (mmol/L)	3.9 (3.7; 4.2)	3.9 (3.7; 4.2)	4.0 (3.6; 4.3)	0.518
Sodium (mmol/L)	136.6 (134.5; 139.8)	136.8 (134.6; 139.9)	135.6 (131.6; 137.8)	0.055
Chloride (mmol/L)	101.9 (98.9; 105.4)	101.9 (99.0; 105.3)	101.1 (98.2; 106.0)	0.840
Bilirubin Total (µmol/L)	10.1 (7.4; 13.2)	10.0 (7.4; 13.2)	11.2 (8.7; 13.1)	0.306
Bilirubin Direct (µmol/L)	2.1 (1.5; 3.1)	2.0 (1.5; 3.0)	2.3 (1.8; 3.7)	0.246
ALT (U/L)	30.0 (16.4; 58.6)	29.8 (16.5; 61.3)	30.3 (13.6; 50.7)	0.495
AST (U/L)	28.9 (19.0; 48.2)	28.5 (18.8; 47.1)	30.4 (19.7; 54.0)	0.617
LDH (U/L)	260.5 (186.7; 360.2)	258.0 (184.0; 360.2)	287.8 (214.6; 397.2)	0.501
Alpha-Amylase (U/L)	52.4 (39.1; 92.6)	53.9 (41.6; 100.6)	41.0 (26.8; 53.1)	0.021
Lactate (mmol/L)	1.2 (0.9; 1.5)	1.1 (0.9; 1.5)	1.4 (1.1; 1.9)	0.137
CRP (mg/L)	43.9 (18.5; 88.3)	41.5 (18.0; 78.2)	79.5 (35.5; 142.7)	0.003
Total Protein (g/L)	62.2 (56.5; 67.9)	62.8 (56.8; 68.2)	58.6 (53.4; 64.8)	0.010
Albumin (g/L)	31.3 (26.3; 35.6)	32.0 (27.3; 35.7)	26.7 (21.4; 31.5)	<0.001
Glucose (mmol/L)	5.4 (4.9; 6.2)	5.4 (4.9; 6.1)	6.0 (4.6; 6.9)	0.086
Procalcitonin (ng/mL)	0.3 (0.1; 1.4)	0.3 (0.1; 0.8)	1.0 (0.1; 5.0)	0.661
Scales (first 48 h in the ICU)				
SOFA	3 (2; 5)	3 (2; 5)	4 (3; 5)	0.052
GCS	10 (8; 13)	10 (8; 13)	9 (6; 11)	0.011
FOUR	13 (11; 15)	13 (11; 16)	12 (8; 14)	0.015

Abbreviations: ALT, alanine aminotransferase; AST, aspartate aminotransferase; BMI, body mass index; CKD, chronic kidney disease; COPD, chronic obstructive pulmonary disease; CRP, C-reactive protein; FOUR, Full Outline of UnResponsiveness score; GCS, Glasgow Coma Scale; LDH, lactate dehydrogenase; RBC, red blood cells; SOFA, Sequential Organ Failure Assessment; WBC, white blood cells.

**Table 2:** Parameters 7 days before ICU discharge or death (survivors vs. non-survivors).

Parameters	All, n = 430	Survivors, n = 387	Non-Survivors, n = 43	*p* (Mann–Whitney/Fisher)
Laboratory parameters				
RBC (10^12^/L)	3.51 (3.08; 3.89)	3.53 (3.12; 3.92)	3.15 (2.78; 3.55)	<0.001
Hemoglobin (g/L)	101 (91; 114)	102 (92; 115)	89 (80; 101)	<0.001
WBC (10^9^/L)	7.5 (5.8; 9.8)	7.4 (5.8; 9.5)	9.6 (6.5; 13.3)	0.019
Neutrophils (10^9^/L)	4.9 (3.5; 7.2)	4.8 (3.4; 6.8)	7.3 (5.0; 12.2)	<0.001
Eosinophils (10^9^/L)	0.2 (0.1; 0.3)	0.2 (0.1; 0.3)	0.1 (0.0; 0.2)	0.005
Basophils (10^9^/L)	0.1 (0.0; 0.1)	0.1 (0.0; 0.1)	0.0 (0.0; 0.1)	<0.001
Lymphocytes (10^9^/L)	1.6 (1.1; 2.0)	1.6 (1.2; 2.1)	0.9 (0.7; 1.3)	<0.001
Platelets (10^9^/L)	322.5 (236; 399)	329 (246; 406)	211 (137; 296)	<0.001
Creatinine (µmol/L)	60.8 (51.0; 75.1)	60.8 (51.2; 75.1)	60.9 (48.6; 82.9)	0.819
Urea (mmol/L)	4.1 (2.9; 6.0)	3.8 (2.8; 5.5)	6.9 (4.9; 13.3)	<0.001
Potassium (mmol/L)	3.9 (3.6; 4.3)	3.9 (3.6; 4.3)	4.0 (3.3; 4.3)	0.705
Sodium (mmol/L)	136.8 (133.8; 138.9)	136.8 (134.0; 138.8)	136.4 (129.6; 144.7)	0.939
Chloride (mmol/L)	101.3 (98.0; 104.2)	101.3 (98.1; 104.2)	100.0 (96.8; 106.2)	0.941
Bilirubin Total (µmol/L)	8.2 (6.4; 10.6)	8.0 (6.2; 10.0)	12.1 (8.8; 15.4)	<0.001
Bilirubin Indirect (µmol/L)	9.3 (7.2; 11.2)	9.3 (7.2; 11.2)	ND	NA
Bilirubin Direct (µmol/L)	1.7 (1.1; 2.3)	1.6 (1.1; 2.1)	3.1 (2.1; 5.8)	<0.001
ALT (U/L)	21.5 (12.5; 38.8)	21.4 (12.4; 36.4)	28.6 (14.7; 70.1)	0.052
AST (U/L)	20.9 (14.8; 33.4)	20.6 (14.8; 31.7)	27.8 (14.5; 84.5)	0.046
LDH (U/L)	196.4 (141.2; 286.3)	196.5 (138.9; 286.3)	188.0 (143.0; 623.9)	0.677
Alpha-Amylase (U/L)	44.5 (33.9; 65.1)	45.4 (36.1; 66.4)	31.0 (23.7; 43.4)	0.011
Lactate (mmol/L)	1.2 (0.9; 1.9)	1.1 (0.9; 1.2)	1.9 (1.3; 2.3)	0.004
CRP (mg/L)	33.1 (10.2; 71.9)	29.4 (8.4; 61.3)	112.1 (61.8; 152.8)	<0.001
Total Protein (g/L)	59.5 (54.3; 65.1)	60.0 (54.8; 65.3)	54.7 (50.2; 61.3)	0.003
Albumin (g/L)	30.4 (26.7; 33.9)	30.7 (27.1; 34.3)	26.6 (22.4; 28.5)	<0.001
Glucose (mmol/L)	4.9 (4.5; 5.6)	4.9 (4.5; 5.6)	4.8 (4.2; 5.7)	0.723
Procalcitonin (ng/mL)	0.4 (0.2; 3.2)	0.3 (0.1; 1.0)	0.9 (0.2; 8.1)	0.113
Scales				
SOFA	3 (2; 4)	3 (2; 4)	6 (3; 9)	<0.001
GCS	11 (9; 15)	11 (9; 15)	14 (10; 15)	0.423
FOUR	15 (13; 16)	15 (12; 16)	14 (14; 16)	0.822
Complications				
Anemia	59 (13.7%)	49 (12.7%)	10 (23.3%)	0.063
Coagulopathy	2 (0.5%)	1 (0.3%)	1 (2.3%)	0.19
Heart Failure	20 (4.7%)	19 (4.9%)	1 (2.3%)	0.708
Pneumonia	181 (42.1%)	158 (40.8%)	23 (53.5%)	0.142
Sepsis	92 (35.7%)	74 (33.3%)	18 (50.0%)	0.062
Septic Shock	12 (2.8%)	4 (1.0%)	8 (18.6%)	<0.001
Polyneuropathy	10 (2.3%)	8 (2.1%)	2 (4.7%)	0.263
Central Nervous System Inflammation	11 (2.6%)	9 (2.3%)	2 (4.7%)	0.303

Abbreviations: ALT, alanine aminotransferase; AST, aspartate aminotransferase; CRP, C-reactive protein; FOUR, Full Outline of UnResponsiveness score; GCS, Glasgow Coma Scale; LDH, lactate dehydrogenase; NA, not applicable; ND, no data; RBC, red blood cells; SOFA, Sequential Organ Failure Assessment; WBC, white blood cells.

## Data Availability

The authors confirm that the data supporting the findings of this study are available within the article and its Supplementary Materials.

## Supplementary Materials

The following supporting information can be downloaded at: https://www.mdpi.com/article/10.3390/jcm14228202/s1, Supplemental S1: STROBE Statement, checklist of items that should be included in reports of cohort studies; Table S1: Results of univariable logistic regression for parameters on admission to the ICU for survivors and non-survivors; Table S2: Results of multivariable regression of parameters on admission to ICU (left-aligned model); Table S3: Results of univariable logistic regression for parameters, relevant at 7 days before discharge from the ICU/death for survivors and non-survivors; Table S4: Results of multivariable regression of parameters relevant at 6–8 days before discharge from ICU (right-aligned model); Table S5: Assessment of right- and left-aligned models.
