# Supplementary material for: Prognostic Modelling of Mortality in Chronic Critical Illness After Traumatic Brain Injury
Source: J Clin Med. 2025 Nov 19;14(22):8202. doi: 10.3390/jcm14228202 (PMC12653882; doi:10.3390/jcm14228202)
Supplement: Supplementary file 1 [file jcm-14-08202-s001.zip › jcm-3943515-supplementary.pdf]

Supplementary Material

Contents

Supplemental S1 – STROBE Statement, checklist of items that should be included in reports of *cohort studies* 1

Table S1. Results of univariable logistic regression for parameters on admission to the ICU for survivors and non-survivors. 4

Table S2. Results of multivariable regression of parameters on admission to ICU (left-aligned model). 6

Table S3. Results of univariable logistic regression for parameters, relevant at 7 days before discharge from the ICU / death for survivors and non-survivors. 7

Table S4. Results of multivariable regression of parameters relevant at 6-8 days before discharge from ICU (right-aligned model). 9

Table S5. Assessment of right- and left-aligned models. 9

Supplemental S1 – STROBE Statement, checklist of items that should be included in reports of *cohort studies*

	Item No	Recommendation	Location
**Title and abstract**	1	(*a*) Indicate the study's design with a commonly used term in the title or the abstract	Abstract
		(*b*) Provide in the abstract an informative and balanced summary of what was done and what was found	Abstract
**Introduction**			
Background/rationale	2	Explain the scientific background and rationale for the investigation being reported	Introduction
Objectives	3	State specific objectives, including any prespecified hypotheses	Introduction
**Methods**			
Study design	4	Present key elements of study design early in the paper	Data acquisition
Setting	5	Describe the setting, locations, and relevant dates, including periods of recruitment, exposure, follow-up, and data collection	Data acquisition
Participants	6	(*a*) Give the eligibility criteria, and the sources and methods of selection of participants. Describe methods of follow-up	Data acquisition
		(*b*) For matched studies, give matching criteria and number of exposed and unexposed	NA
Variables	7	Clearly define all outcomes, exposures, predictors, potential confounders, and effect modifiers. Give diagnostic criteria, if applicable	Data acquisition
Data sources/ measurement	8*	For each variable of interest, give sources of data and details of methods of assessment (measurement). Describe comparability of assessment methods if there is more than one group	Data acquisition

Bias	9	Describe any efforts to address potential sources of bias	Data acquisition
Study size	10	Explain how the study size was arrived at	NA (whole database analysis)
Quantitative variables	11	Explain how quantitative variables were handled in the analyses. If applicable, describe which groupings were chosen and why	Statistical analysis
Statistical methods	12	(*a*) Describe all statistical methods, including those used to control for confounding	Statistical analysis
		(*b*) Describe any methods used to examine subgroups and interactions	Statistical analysis
		(*c*) Explain how missing data were addressed	Statistical analysis
		(*d*) If applicable, explain how loss to follow-up was addressed	NA
		(*e*) Describe any sensitivity analyses	Statistical analysis
**Results**			
Participants	13*	(a) Report numbers of individuals at each stage of study—eg numbers potentially eligible, examined for eligibility, confirmed eligible, included in the study, completing follow-up, and analysed	Figure 1
		(b) Give reasons for non-participation at each stage	Figure 1
		(c) Consider use of a flow diagram	Figure 1
Descriptive data	14*	(a) Give characteristics of study participants (eg demographic, clinical, social) and information on exposures and potential confounders	Table 1
		(b) Indicate number of participants with missing data for each variable of interest	Table 1
		(c) Summarise follow-up time (eg, average and total amount)	NA
Outcome data	15*	Report numbers of outcome events or summary measures over time	Table 1
Main results	16	(*a*) Give unadjusted estimates and, if applicable, confounder-adjusted estimates and their precision (eg, 95% confidence interval). Make clear which confounders were adjusted for and why they were included	Table 2; Figure 3; Tables S1-S5
		(*b*) Report category boundaries when continuous variables were categorized	Table S5
		(*c*) If relevant, consider translating estimates of relative risk into absolute risk for a meaningful time period	NA
Other analyses	17	Report other analyses done—eg analyses of subgroups and interactions, and sensitivity analyses	NA
**Discussion**			
Key results	18	Summarise key results with reference to study objectives	Key findings
Limitations	19	Discuss limitations of the study, taking into account sources of potential bias or imprecision. Discuss both direction and magnitude of any potential bias	Strengths and limitations
Interpretation	20	Give a cautious overall interpretation of results considering objectives, limitations, multiplicity of analyses, results from similar studies, and other relevant evidence	Conclusion

Generalisability	21	Discuss the generalisability (external validity) of the study results	Significance of study findings
**Other information**			
Funding	22	Give the source of funding and the role of the funders for the present study and, if applicable, for the original study on which the present article is based	Meta data

*Give information separately for exposed and unexposed groups.

Note: An Explanation and Elaboration article discusses each checklist item and gives methodological background and published examples of transparent reporting. The STROBE checklist is best used in conjunction with this article (freely available on the Web sites of PLoS Medicine at http://www.plosmedicine.org/, Annals of Internal Medicine at http://www.annals.org/, and Epidemiology at http://www.epidem.com/). Information on the STROBE Initiative is available at http://www.strobe-statement.org.

Table S1. Results of univariable logistic regression for parameters on admission to the ICU for survivors and non-survivors.

Parameters	p (Log regression) continuous
Sex, male	0.586
Age, years	0.027
BMI	0.360
Comorbidities	
Atrial Fibrillation	0.119
Coronary Artery Disease	0.001
Valvular Heart Disease	0.466
Arterial Hypertension	0.265
Type 2 Diabetes	0.919
Type 1 Diabetes	0.339
CKD	0.003
COPD	>0.9
Polytrauma	0.732
Multiorgan Failure on Admission	0.006
Malignant Tumor	>0.9
Laboratory parameters (first 48 h in the ICU)	
RBC (10^{12}/L)	0.012
Hemoglobin (g/L)	0.001
WBC (10^{9}/L)	0.537
Neutrophils (10^{9}/L)	0.509
Eosinophils (10^{9}/L)	0.130
Basophils (10^{9}/L)	0.052
Lymphocytes (10^{9}/L)	0.653
Platelets (10^{9}/L)	0.265
Creatinine (µmol/L)	0.257
Urea (mmol/L)	0.301
Potassium (mmol/L)	0.644
Sodium (mmol/L)	0.470
Chloride (mmol/L)	0.152
Bilirubin Total (µmol/L)	0.933
Bilirubin Direct (µmol/L)	0.942
ALT (U/L)	0.177
AST (U/L)	0.645
LDH (U/L)	0.473
Alpha-Amylase (U/L)	0.200
Lactate (mmol/L)	0.498
CRP (mg/L)	0.002
Total Protein (g/L)	0.019
Albumin (g/L)	<0.001
Glucose (mmol/L)	0.364
Procalcitonin (ng/mL)	0.777
Scales (first 48 h in the ICU)	
SOFA	0.102

GCS	0.006
FOUR	0.004

Abbreviations: ALT, alanine aminotransferase; AST, aspartate aminotransferase; BMI, body mass index; CI, confidence interval; CKD, chronic kidney disease; COPD, chronic obstructive pulmonary disease; CRP, C-reactive protein; FOUR, Full Outline of UnResponsiveness score; GCS, Glasgow Coma Scale; LDH, lactate dehydrogenase; OR, odds ratio; RBC, red blood cells; SOFA, Sequential Organ Failure Assessment; WBC, white blood cells.

Table S2. Results of multivariable regression of parameters on admission to ICU (left-aligned model).

Step	Parameter	p	OR (95% CI)
8	Coronary Artery Disease	0.023	2.914 (1.160; 7.316)
	Multiorgan Failure on Admission	0.035	3.878 (1.101; 13.659)
	CRP (mg/L)	0.044	1.005 (1.000; 1.009)

Note: Variables entered at Step 1: age, coronary artery disease, CKD, multiorgan failure on admission, hemoglobin, CRP, total protein, albumin, GCS, FOUR.
Abbreviations: CI, confidence interval; CKD, chronic kidney disease; CRP, C-reactive protein; FOUR, Full Outline of UnResponsiveness; GCS, Glasgow Coma Scale; OR, odds ratio.

Table S3. Results of univariable logistic regression for parameters, relevant at 7 days before discharge from the ICU / death for survivors and non-survivors.

Parameters	p (Log regression) continuous
Sex, male	0.586
Age, years	0.027
BMI	0.360
Comorbidities	
Atrial Fibrillation	0.119
Coronary Artery Disease	0.001
Valvular Heart Disease	0.466
Arterial Hypertension	0.265
Type 2 Diabetes	0.919
Type 1 Diabetes	0.339
CKD	0.003
COPD	>0.9
Polytrauma	0.732
Multiorgan Failure on Admission	0.006
Malignant Tumor	>0.9
Laboratory parameters (6-8 days before discharge from ICU / death)	
RBC (10^{12}/L)	<0.001
Hemoglobin (g/L)	<0.001
WBC (10^9/L)	0.003
Neutrophils (10^9/L)	<0.001
Eosinophils (10^9/L)	0.056
Basophils (10^9/L)	0.001
Lymphocytes (10^9/L)	<0.001
Platelets (10^9/L)	<0.001
Creatinine (µmol/L)	0.006
Urea (mmol/L)	<0.001
Potassium (mmol/L)	0.226
Sodium (mmol/L)	0.100
Chloride (mmol/L)	0.073
Bilirubin Total (µmol/L)	<0.001
Bilirubin Indirect (µmol/L)	NA
Bilirubin Direct (µmol/L)	0.008
ALT (U/L)	0.009
AST (U/L)	0.001
LDH (U/L)	0.168
Alpha-Amylase (U/L)	0.409
Lactate (mmol/L)	0.018
CRP (mg/L)	<0.001
Total Protein (g/L)	0.002
Albumin (g/L)	<0.001
Glucose (mmol/L)	0.344
Procalcitonin (ng/mL)	0.291
Scales (6-8 days before discharge from ICU / death)	

SOFA	<0.001
GCS	0.437
FOUR	0.458
Complications	
Anemia	0.060
Coagulopathy	0.119
Heart Failure	0.456
Pneumonia	0.113
Sepsis	0.056
Septic Shock	<0.001
Polyneuropathy	0.300
Central Nervous System Inflammation	0.369

Abbreviations: ALT, alanine aminotransferase; AST, aspartate aminotransferase; BMI, body mass index; CI, confidence interval; CKD, chronic kidney disease; COPD, chronic obstructive pulmonary disease; CRP, C-reactive protein; FOUR, Full Outline of UnResponsiveness score; GCS, Glasgow Coma Scale; LDH, lactate dehydrogenase; OR, odds ratio; RBC, red blood cells; SOFA, Sequential Organ Failure Assessment; WBC, white blood cells.

Table S4. Results of multivariable regression of parameters relevant at 6-8 days before discharge from ICU (right-aligned model).

Step	Parameter	p	OR (95% CI)
9	Lymphocytes (10^9/L)	0.007	0.217 (0.072; 0.659)
	Platelets (10^9/L)	0.046	0.995 (0.990; 1.000)
	Urea (mmol/L)	0.005	1.243 (1.066; 1.449)
	CRP (mg/L)	0.022	1.010 (1.001; 1.019)

Note: Variables entered at Step 1: age, hemoglobin, WBC, neutrophils, lymphocytes, platelets, urea, bilirubin total, ALT, AST, CRP, albumin.
Abbreviations: CI, confidence interval; CKD, chronic kidney disease; CRP, C-reactive protein; FOUR, Full Outline of UnResponsiveness; GCS, Glasgow Coma Scale; OR, odds ratio.

Table S5. Assessment of right- and left-aligned models.

Parameter	AUROC (95% CI)	p	Cut-off	Se (95% CI)	Sp (95% CI)	PPV (95% CI)	NPV (95% CI)	OR 95% CI	RR 95% CI
Score on admission	0.720 (0.631; 0.808)	<0.001	8.379	58.8 (40.7; 75.4)	75.7 (69.9; 80.8)	24.4 (18.5; 31.5)	93.2 (90.2; 95.4)	4.447 (2.121; 9.325)	3.606 (1.914; 6.794)
Score 6-8 days	0.889 (0.831; 0.947)	<0.001	12.277	90.0 (73.5; 97.9)	74.9 (69.4; 79.9)	27.8 (23.4; 32.8)	98.6 (96.0; 99.5)	26.871 (7.908; 91.305)	19.670 (6.115; 63.274)

Abbreviations: AUROC, area under the receiver operating characteristic curve; CI, confidence interval; NPV, negative predictive value; OR, odds ratio; PPV, positive predictive value; RR, relative risk; Se, sensitivity; Sp, specificity.
